# Numerical and experimental study on the relationship between pull-out force and indentation depth of aviation wire crimp terminal

**DOI:** 10.1038/s41598-022-26563-7

**Published:** 2022-12-19

**Authors:** Pin Li, Gongping Liu, Ping Wang, Guangwen Huang, Zhuonan Yu, Hang Xiu, Chunlin Tian

**Affiliations:** 1 School of Mechanical and Electrical Engineering, Changchun University of Science and Technology, Changchun, 130022 China; 2 Changchun University of Science and Technology Chongqing Research Institute, Chongqing, 401135 China; 3 AVIC Xi’an Aircraft Industry (Group) Company LTD, Xi’an, 710089 China

**Keywords:** Engineering, Mechanical engineering

## Abstract

To study the numerical relationship between the pull-out force and indentation depth of aviation wire crimp terminal, the crimping process between electrical contacts and stranded conductors and the tensile process of crimping assembly were simulated by the explicit dynamic finite element method. Regarding the variation trend of the tension of the crimping assembly with the tensile displacement during the tensile process and the failure mode, the numerical results and the experimental results showed a high degree of fit, and the relative error of the pull-out force was only 2.6%, which verified the reliability of the established numerical model. This model obtained the pull-out force curve of the crimp terminal that changes with the indentation depth. The authors suggest selecting the interval where the pull-out force is not less than 95% of the peak value, and the depth is less than the corresponding value at the peak value as the best value range of the indentation depth.

## Introduction

**Figure 1:**
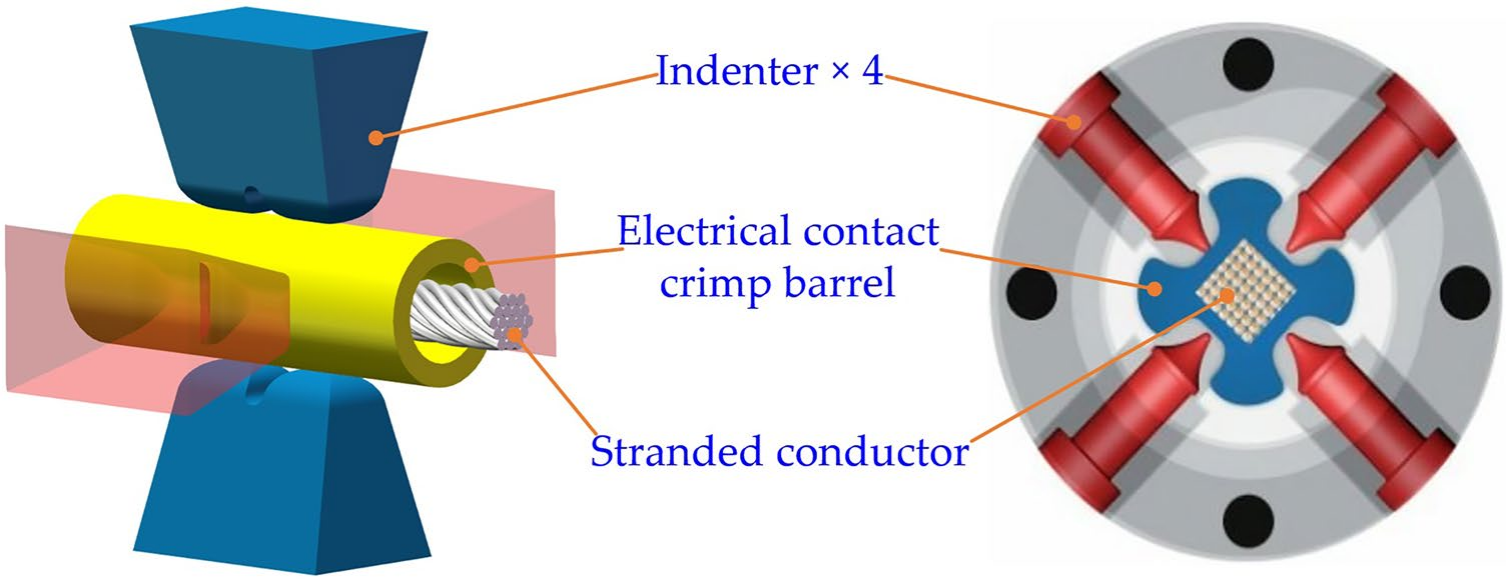
Four-indenter-eight-indentation crimping method for aviation electrical contacts.

The reliability requirements of aviation wire crimp terminals are extremely high and demanding. Once they fail, the huge losses caused will be irreparable. In the failure rate statistics of American aerospace, the proportion of electrical connector failures is as high as 10%, and about 50–60% of them are directly caused by the failure of the electrical performance of the crimp terminal^1–3^. The connection of dissimilar metal materials has always been a difficult problem in the industry. Different from the more commonly used methods such as riveting and adhesive bonding^4,5^, the electrical connection between aviation electrical contacts and wire conductors mainly adopts the four-indenter-eight-indentation crimping method shown in Fig. 1. The evaluation of the electrical performance of the crimp terminal is mainly reflected in two aspects: the pull-out force and the local voltage drop^6,7^. Their measured values are mainly related to three independent factors: the crimping component material, the indenter shape, and the indentation depth^8^. In recent years, in the face of the increasingly diverse characteristics of crimping component materials, the difference in indentation depth required for specific combinations of wires and electrical contacts of the same size and different materials has become more prominent. Traditional crimping tools have been unable to meet aviation wire ends' high-quality, multi-adaptability processing requirements. Therefore, it is essential to explore the method of determining the optimal indentation depth required for a specific combination of electrical contacts and wires to develop intelligent crimping equipment.

At present, the exploration of the crimping mechanism between electrical contacts and wires is mainly based on two methods: theoretical derivation and numerical simulation. For the former, in the previous work^9^ of the author of this paper, the mathematical model of the relationship between the pull-out force and the indentation depth of the aviation wire crimp terminal was derived from a 2D perspective. Within a reasonable indentation depth range, the pullout resistance values exhibited by the model are in good agreement with the experimental results. However, the model does not apply when the terminal is in an over-crimped state, which results in the model being unable to represent the optimal indentation depth for the crimping group numerically directly. Compared with theoretical derivation, the numerical simulation method is more prevalent in many types of research because of its simple and intuitive characteristics.

A high capability of computer analysis and modeling is usually required to simulate the elastic–plastic deformation process. Therefore, to simplify the calculation cost, the initial more research work tends to simulate the dynamic crimping process between the electrical contact and the wire conductor from a 2D perspective. Among them, Kugener^10^ took the lead in simulating the crimping process of open-barrel electrical contacts based on the plane stress assumption method by creating a deformable body to represent the multi-core conductor and its sheath as a whole. Due to the existence of gaps inside the actual multi-core conductor, to keep the simulation results consistent with the actual ones, Young's modulus of the imaginary conductor has to be reduced for reference compared to the real conductors. Compared with Kugener’s work, Morita^11^ carried out the crimping simulation based on the plane strain assumption method. The author found that the simulated crimping force would be greatly increased under this method. Abbas^12^ pointed out in his paper that the plane strain assumption method is unsuitable for crimping simulation because the possibility of the material being extruded out of the plane must be ignored in the simulation process. In the study of the four-point crimping method, Lorrière^13^ compared the above two 2D plane hypothetical simulations with 3D simulations and concluded that the 3D simulation is the only correct method to simulate crimping. Mocellin^14^, Petitprez^15^, and others conducted 2D and 3D simulations of the crimping process of aviation closed-barrel electrical contacts for the first time. Through comparison with experiments, they pointed out that the 2D method can only give approximate results, and ignoring the plastic flow of the crimped material in the third direction would lead to an over-constrained model.

Crimping is a quasi-static but highly nonlinear problem, and its corresponding time-integral solving algorithms in simulation usually include two types: implicit and explicit. Since the crimping process involves complex multi-body contact between the crimping barrel and the wire conductor and elastic–plastic deformation of the components, the implicit algorithm usually shows lower efficiency for this situation, so many scientists prefer the explicit algorithm^10–13,16–19^. Among them, Zhmurkin^16,17^ used the 3D nonlinear explicit dynamic finite element method to numerically simulate the open-barrel crimping process of 7-core stranded conductors, the effects of the elastic spring-back the components after unloading and geometric parameters of the serrated structure of the crimping barrel on the crimping quality were studied. To study the effects of design variables such as crimping height, crimping width, and crimping die shape on the compression ratio of multi-core stranded conductors, Kim et al.^18,19^ carried out 2D and 3D explicit finite element simulations of the crimping process of open-barrel electrical contacts. It is worth noting that the study of Petitprez et al.^20–23^, on the premise that they have been convinced that the explicit finite element model can better solve the multi-domain contact problem, the finite element software based on an implicit algorithm was used to inverse analyze the performance parameters of the crimping material. Then, based on this parameter, the same software was used to simulate the crimping of aviation electrical contacts and the tensile process of the crimping assembly. The results show that although the calculation time is very long, this method can also effectively predict the effect of the performance parameters, size, and indentation depth of the crimping material on the mechanical properties of the crimp terminal. Nevertheless, to realize the accurate production of high-quality aviation wire crimp terminals by a new generation of crimping equipment, the specific numerical relationship between the pull-out force and indentation depth of the crimp terminal still needs to be deeply studied.

In this paper, based on the explicit dynamic finite element technology, the numerical simulation of the crimping process between aviation electrical contacts and wires and the tensile process of the crimping assembly was carried out. In terms of the shape, pull-out force, and tensile failure mode of the crimp terminal, the numerical results were compared with the experimental results to verify the reliability of the established numerical model. We further studied the specific numerical relationship between the pull-out force and the indentation depth of the crimp terminal. The selection criteria for the optimal indentation depth range were analyzed and given.

## Dynamic finite element simulation theoretical background

### Implicit and explicit algorithms applied to models

The implicit algorithm is generally applied to the analysis of static and quasi-static problems, and its time integral form at time *t* can be expressed as follows:

1 $$\left[M\right]\left\{{a}_{t}\right\}+\left[C\right]\left\{{v}_{t}\right\}+\left[K\right]\left\{{u}_{t}\right\}=\left\{{F}_{t}\right\} ,$$

where [*M*] is the mass matrix, [*C*] is the damping matrix, [*K*] is the stiffness matrix, {${a}_{t}$} is the acceleration vector, {${v}_{t}$} is the velocity vector, {${u}_{t}$} is the displacement vector, and {${F}_{t}$} is the external force vector. If inertial effects ([*C*] and [*M*]) are not considered, at time *t* + *Δt*, Eq. (1) can be expressed as follows:

2 $$\left\{{u}_{t+\Delta t}\right\}={\left[K\right]}^{-1}\left\{{F}_{t+\Delta t}\right\}.$$

For nonlinear problems, Eq. (2) can be solved for displacement and average external force at time *t* + *Δt* by a series of linear approximations (*Newton–Raphson* iterations) based on small time steps. However, for highly nonlinear problems, implicit algorithms often fail to guarantee solution convergence.

The explicit algorithm generally first obtains the acceleration value at time *t*, and then further obtains the velocity and displacement value of the corresponding time step based on the central difference method. The explicit time integration form is as follows:

3 $$\left\{{a}_{t}\right\}={\left[M\right]}^{-1}\left(\left[{F}_{t}^{ext}\right]-\left[{F}_{t}^{int}\right]\right) ,$$

where ${F}_{t}^{ext}$ is the vector of applied external force and physical force, and ${F}_{t}^{int}$ is the internal force vector.

The velocity and displacement of nodes can be obtained by the following Eqs. (4) and (5), respectively:

4 $$\left\{{v}_{t+\Delta t/2}\right\}=\left\{{v}_{t-\Delta t/2}\right\}+\left\{{a}_{t}\right\}\Delta {t}_{t} ,$$

5 $$\left\{{u}_{t+\Delta t}\right\}=\left\{{u}_{t}\right\}+\left\{{v}_{t+\Delta t/2}\right\}\Delta {t}_{t+\Delta t/2} ,$$

where $\Delta {t}_{t+\Delta t/2}=0.5\left(\Delta {t}_{t}+\Delta {t}_{t+\Delta t}\right)$. Then, the new geometric configuration can be obtained by adding the displacement increment to the initial configuration, as follows:

6 $$\left\{{x}_{t+\Delta t}\right\}=\left\{{x}_{0}\right\}+\left\{{u}_{t+\Delta t}\right\}.$$

For nonlinear analyses, although the explicit algorithm does not require convergence checks, smaller time steps are still necessary for the computation to remain stable. In general, the time step for which the computation is guaranteed to converge must satisfy the following equation:

7 $$\Delta t\le \Delta {t}_{cr}=\frac{2}{{\omega }_{\mathrm{max}}},$$

where ${\omega }_{\mathrm{max}}$ is the highest natural vibration frequency of the system, and its value is generally determined by the smallest element in the corresponding configuration mesh at each moment.

### Correction of stress–strain function

Typically, the nominal stress–strain function can be obtained by standard tensile or compressive experiments as follows:

8 $${\sigma }_{e}=\frac{F}{{S}_{0}}=f\left({\varepsilon }_{e}\right) ,$$

where ${\sigma }_{e}$ is the nominal stress, ${\varepsilon }_{e}$ is the nominal strain, ${\varepsilon }_{e}=\left(L-{L}_{0}\right)/{L}_{0}$, ${L}_{0}$ is the initial length, *F* is the normal force, and ${S}_{0}$ is the initial cross-sectional area. However, when the material is in a state of non-uniform plastic deformation, local necking will occur, resulting in an inconsistent cross-sectional area of the overall material. Therefore, the nominal stress–strain function is reasonably accurate for small deformation analysis but unacceptable for realistic, in-depth mechanical engineering analysis.

Facing the above problems, Francois et al.^24^ corrected the nominal stress–strain function based on the first-order approximation by assuming that the material volume is conserved. The nominal values of stress and strain measured by experiments are converted into real values that can be used in practical engineering problems. The correction equations are as follows:

9 $${\sigma }_{t}={\sigma }_{e}\left(1+{\varepsilon }_{e}\right)$$

10 $${\varepsilon }_{t}=ln\left(1+{\varepsilon }_{e}\right),$$

where ${\sigma }_{t}$ and ${\varepsilon }_{t}$ are the true stress and strain values at time t, respectively.

However, it is very difficult to obtain the completely true stress–strain relationship function of materials in practical engineering. To simplify the simulation workload, usually, we can first use the above correction equations to convert the material properties such as yield strength ${\sigma }_{s}$, tensile strength ${\sigma }_{b}$, Young’s modulus *E*, elongation *δ* provided by the manufacturer into the true yield strength ${\sigma }_{s\_t}$, yield strain ${\varepsilon }_{s\_t}$, tensile strength ${\sigma }_{b\_t}$, and tensile strain ${\varepsilon }_{b\_t}$. Then, the bilinear estimation method is used to simplify the true stress–strain relationship function of the material. The first stage of the bilinear is elastic, and the slope is Young's modulus *E*; the second stage is plastic, and the slope is the tangent modulus ${E}_{T}$, and its value can be obtained by the following equation:

11 $${E}_{T}=\frac{{\sigma }_{b\_t}-{\sigma }_{s\_t}}{{\varepsilon }_{b\_t}-{\varepsilon }_{s\_t}}$$

### Definition of contact interface

In dynamic finite element simulation, there are usually three types of model interface contact: single-face, point-to-face, and face-to-face. Among them, the single-face contact type is the most popular for situations where the model is cumbersome and the contact state is unpredictable. The reason for this is that with this type when the program checks whether the models penetrate each other, all outer surfaces are within the search range, so there is no need to specifically define the contact surface and target surface where contact may occur.

The good handling of the contact interface is inseparable from a reasonable contact control algorithm. For common sliding contacts, the “symmetric penalty function” method is the most commonly used algorithm. The basic principle is that, based on a contact search algorithm and a dual-loop master–slave algorithm for determining normal and tangential forces^25^, the program sequentially verifies the contact compatibility of each node in the model at each time step. Because the algorithm has symmetry and does not require collision and release conditions, it rarely causes the hourglass effect and can accurately ensure the conservation of momentum of the system.

In the dynamic finite element simulation, the friction coefficient ${\mu }_{c}$ is composed of the static friction coefficient ${\mu }_{s}$, the dynamic friction coefficient ${\mu }_{d}$, and the exponential decay coefficient *β*. It is considered that with the increase of the relative velocity ${V}_{rel}$ of the contact surface, the value of the friction coefficient ${\mu }_{c}$ decays exponentially from ${\mu }_{s}$ to ${\mu }_{d}$, as shown in the following equation:

12 $${\mu }_{c}={\mu }_{d}+\left({\mu }_{s}-{\mu }_{d}\right){e}^{-\beta \cdot {V}_{rel}}.$$

In addition, the maximum friction force ${F}_{\mathrm{max}}$ can be defined by the viscosity coefficient ${V}_{c}$:

13 $${F}_{max}={V}_{c}\cdot {A}_{cont} ,$$

where ${A}_{cont}$ is the contact area, it is recommended that $V\_c=\sigma \_s/\sqrt 3$, and ${\sigma }_{s}$ is the yield stress of the contacting material.

### Dynamic Saint–Venant’s principle

Numerous studies have confirmed the application of Saint–Venant’s principle (SVP) to dynamic problems. For the dynamic finite element simulation of nonlinear metal local forming, the calculation results should fully consider the boundary effect based on SVP. Karp^26^ gave a dynamic interpretation of the SVP and believed that whether the structure satisfies the dynamic SVP depends on the following two conditions:

1. The dynamic resultant force of the external load is zero;
2. The stress wave excited by the external load in the structure has zero effect on the far end of the system.

The energy flow carried by the stress wave propagating in space is generally considered a measure of wave intensity. The derivation of the energy flow can be expressed as follows^27^:

14 $$P=\frac{1}{2}Gd\xi \frac{{\omega }^{3}}{{C}_{T}}{A}^{2} ,$$

where, *P* is the energy flow, *G* is the shear modulus, *d* is the thickness of the propagation medium, *ξ* is the wave number, *ω* is the frequency, *A* is the amplitude, and *C*_*T*_ is the phase velocity of the shear wave.

## Pre-processing of aviation electrical contact crimping simulation

**Figure 2:**
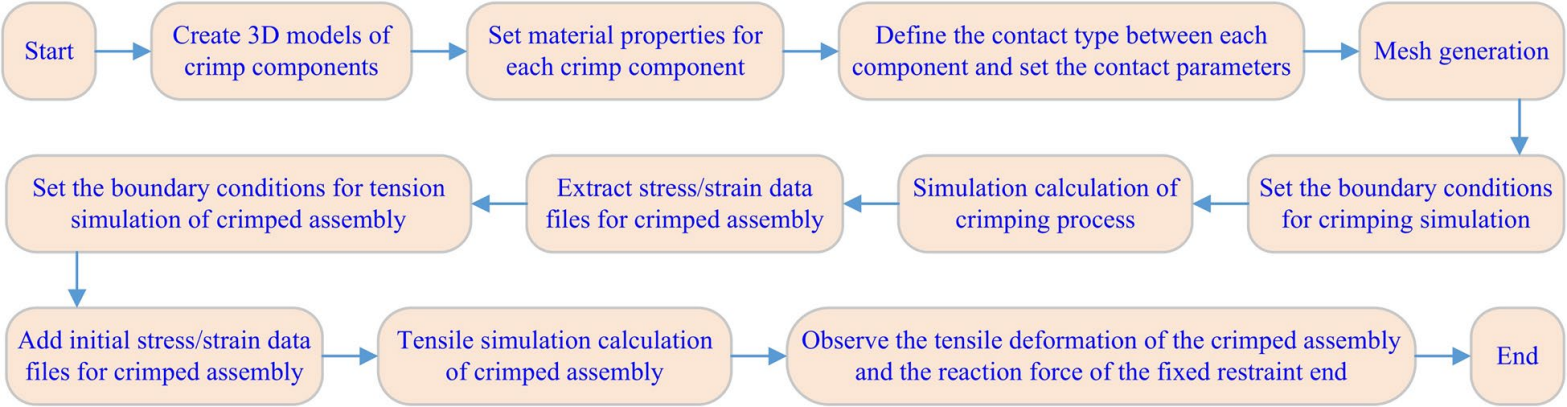
The flow of the overall simulation work.

In this study, we used the LS-DYNA module under the CAE software ANSYS™ to calculate the crimping process and the crimped assembly's tensile process. LS-DYNA is a nonlinear dynamic finite element analysis program based on explicit algorithms, which is especially suitable for solving nonlinear metal forming problems in quasi-static mode. Figure 2 shows the flow of the overall simulation. Next, let's introduce the pre-processing work of crimping simulation.

### Crimping model and material properties

**Figure 3:**
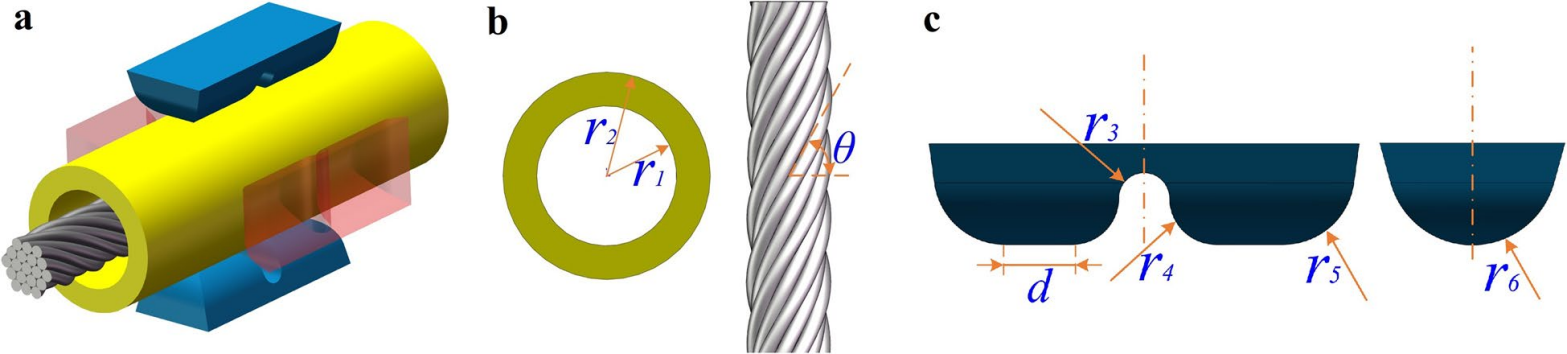
3D crimping simulation model construction and dimension setting. (**a**) 3D crimping simulation model; (**b**) electrical contacts and stranded conductors; (**c**) crimping action part of the indenter.

Figure 3A shows the overall 3D simulation model, including the indenter. Unlike previous crimping simulation studies, the stranded conductor model preserves the twisting characteristics of actual multi-core strands. Even though this would prevent the overall model from being symmetrically simplified, the simulation results would be more realistic.

According to the adaptation requirements of the size specification between the electrical contact and the stranded conductor in the standard *SAE-AS39029*^28^ document, 20*#* electrical contacts and 22*AWG* 19-core stranded conductors were selected as the basis for dimensioning the crimping component model. As shown in Fig. 3b, the diameter of a single core in the strand is 0.15* mm*, the twist angle *θ* is 60°, the inner surface radius ${r}_{1}$ of the electrical contact crimping barrel is 0.6* mm*, and the outer surface radius ${r}_{2}$ is 0.89* mm*. Figure 3(c) shows the morphology of the crimping part of the indenter, and Table 1 lists the relevant design dimensions according to the design specification requirements of the stamper in the standard *SAE-AS22520* document^29^.

**Table 1:** Relevant design dimensions of the crimping action part of the indenter (*mm*).

${r}_{3}$	${r}_{4}$	${r}_{5}$	${r}_{6}$	*d*
0.15	0.28	0.42	0.5	0.4

According to the regulations on the material properties of crimping components in the standard *SAE-AS39029* document, the material for the crimping barrel of the aviation electrical contact is C17200 beryllium copper, and the material for the aviation stranded conductor is traditional oxygen-free copper. Their density *ρ*, Young's modulus *E*, Poisson's ratio *v*, yield strength ${\sigma }_{0.2}$, tensile strength ${\sigma }_{b}$, and elongation *δ* are listed in Table 2, respectively. In addition, the stamper is generally made of cemented carbide steel material with high hardness, high bearing capacity, and strong wear resistance, which has an excellent ability to resist deformation. Therefore, in the process of crimping simulation, the stamper was treated as a rigid object to simplify the calculation.

**Table 2:** Relevant mechanical properties of crimping components.

	*ρ* ($\mathrm{g}\cdot {cm}^{-3}$)	*E* (MPa)	${\sigma }_{0.2}$(MPa)	${\sigma }_{b}$(MPa)	*δ* (%)	*v*
Crimping barrel	8.3	1.28 × 105	483	552	25	0.35
Stranded conductor	8.96	1.24 × 105	90	200	30	0.34

### Contact definition and meshing

The crimping of aviation electrical contacts is a multi-domain extrusion contact problem, and the possible contact positions are relatively ambiguous. Therefore, the overall model adopted the single-face contact type introduced in Section “Definition of Contact Interface”, avoiding defining unknown contact surfaces and target surfaces. In addition, for the extrusion contact problem, mutual penetration between the model surface nodes is not allowed during the simulation process. Using the "symmetric penalty function" method introduced in Section “Definition of Contact Interface” as the contact control algorithm for the overall model can effectively solve this problem. At the same time, the algorithm can also significantly reduce the zigzag contact deformation caused by the hourglass effect^30^. Referring to the research of Kim et al.^19^, the static friction coefficient of the crimping barrel-wire core contact, the crimping barrel-indenter contact, and the crimping barrel self-contact is set to 0.2. The static friction coefficient of the contact between the wire cores is set to 0.15, the dynamic friction coefficient of all contacts is set to 0.1, and the decay coefficient is set to 1 by default.

**Figure 4:**
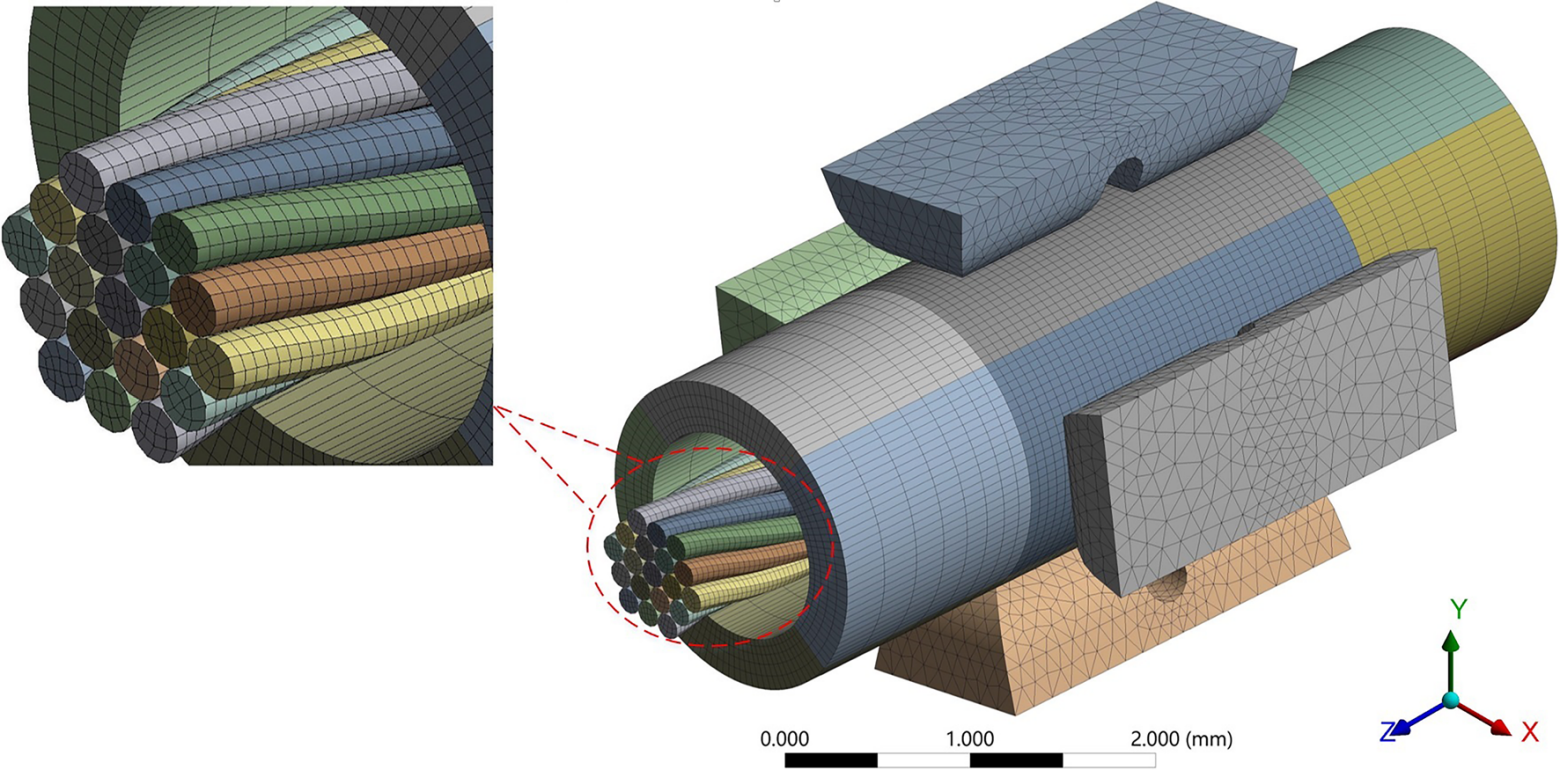
Meshing of the crimping model.

As shown in Fig. 4, the overall model adopted two meshing methods. Applying a tetrahedral mesh to indenter models with relatively complex structural features will make it easier to automatically refine the mesh in critical areas using curvature and approximate size functions. Applying a hexahedral mesh at the same density as the tetrahedral mesh will exhibit fewer elements and nodes. Therefore, we used the hexahedral mesh to the crimping barrel and stranded conductor models based on the sweep method to obtain a more regular and uniform mesh distribution. In addition, considering the calculation accuracy, it is necessary to perform mesh refinement on the indenter-crimping barrel contact area and the crimping barrel-stranded conductor contact area. At the same time, to balance calculation accuracy and time, the rationality of mesh refinement needs to be verified. Generally, a mesh-independent solution is considered to be obtained when the mesh is refined to have a negligible effect on the results. Here, we set five groups of minimum element size for the above two mesh refinement areas, and Table 3 shows the number of elements corresponding to each group.

**Table 3:** The number of elements corresponding to different mesh refinement densities.

Contact area	Minimum element size (*mm*)				
Indenter-crimping barrel	0.04	0.05	0.06	0.07	0.08
Crimping barrel-stranded conductor	0.03	0.035	0.04	0.045	0.05
**Number of elements (thousand)**	≈240	≈160	≈110	≈85	≈70

### Boundary conditions and simulation parameter settings

**Figure 5:**
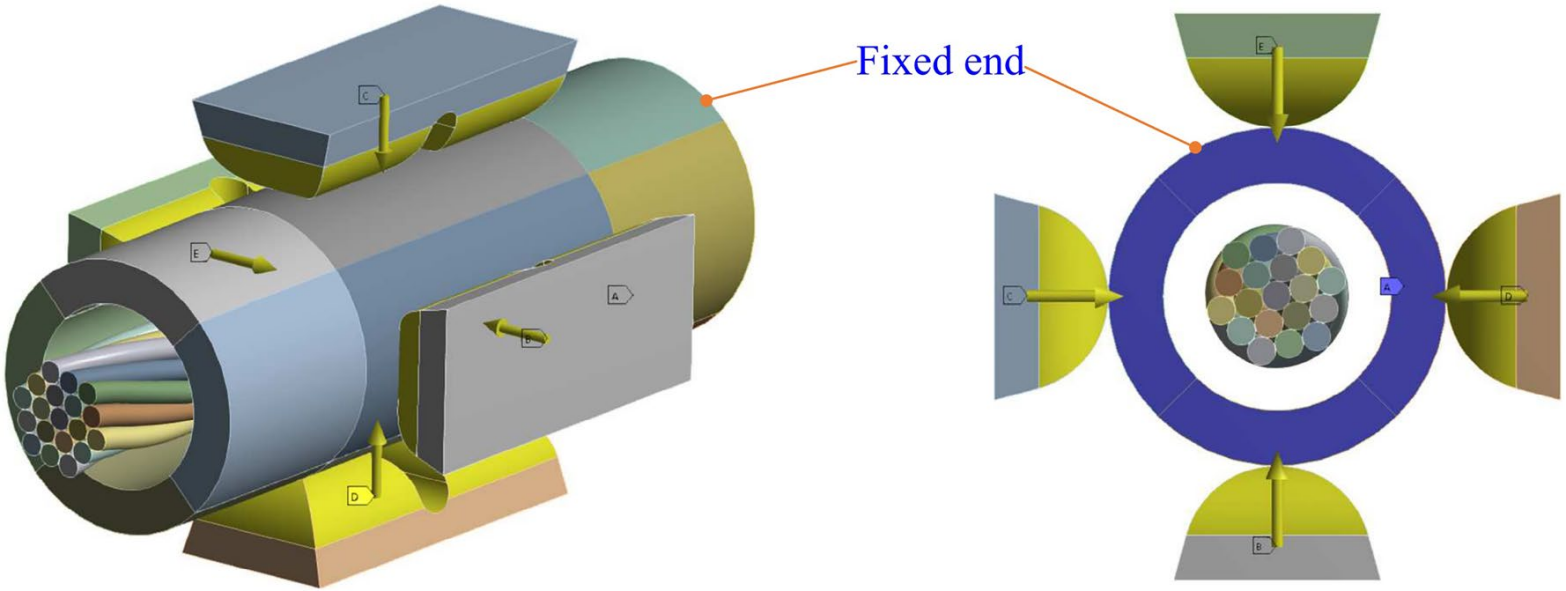
Boundary condition settings for the crimping simulation.

As shown in Fig. 5, one end of the crimping barrel model was fixed according to the actual crimping operation. The four indenters were set to move vertically and centripetally synchronously. We should establish the moving speed of the indenter concerning the time step of the simulation calculation, which is determined by the size of the mesh element and the longitudinal acoustic wave velocity in the model material^31,32^. As mentioned in Section “Implicit and explicit algorithms applied to models”, to obtain numerical stability, the default time step of the system is generally small, which is very unfavorable to the computational efficiency of the simulation of low-speed phenomena such as most metal forming processes. Artificially increasing the material’s mass density or the model's mesh size can increase the time step and speed up the analysis process^33,34^, but this will also reduce the accuracy of the simulation. Reference^10^ pointed out that when the inertia effect is negligible, the speed of the die can be increased to speed up the simulation analysis. Typically for an element size of 0.01* mm*, the default time step *Δt* is about 10^–9^ s. However, for the crimping process considered in this study, the actual indenter speed is about 0.2 m*/s*, which is very slow for the default time step. Increasing the indenter velocity in the simulation to 1 m*/s* did not result in significant inertial effects.

On the other hand, to improve computational efficiency, the explicit dynamic analysis program usually automatically simplifies the integration unit locally to reduce the number of integration points. However, the simplified integration unit is prone to produce zero-energy modes, which lead to invalid calculation results, namely hourglass modes. Studies have pointed out that for the elastic–plastic deformation problem under solid elements, the “*Belytschko-Bindeman*” method can effectively control the hourglass deformation phenomenon that may occur in the simulation^35,36^. For the elastic–plastic crimp deformation process considered here, the hourglass control factor under this method is set to 0.01 by default.

## Experiments

### Crimping of electrical contacts

**Figure 6:**
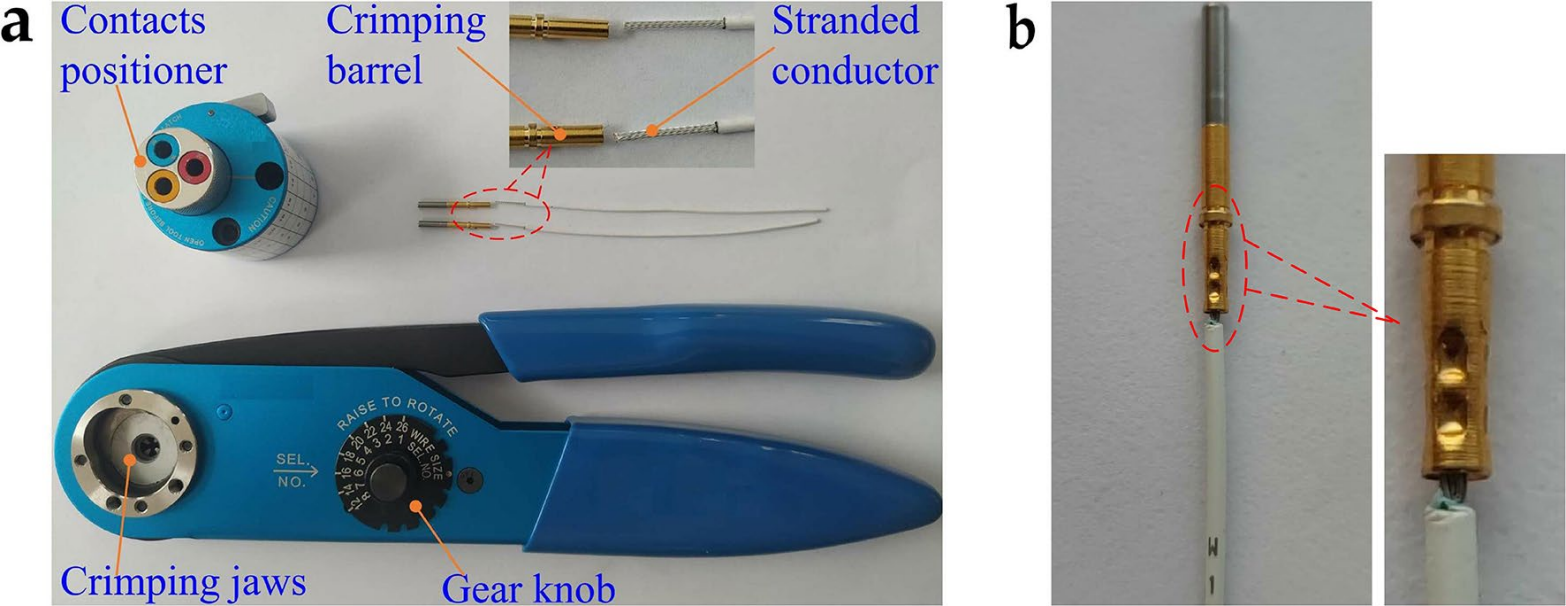
Crimping tools, materials, and crimping samples.

Figure 6A shows the tools and materials used for the crimping experiment. Among them, the electrical contact is a 20*#* gold-plated beryllium copper alloy contact suitable for *MIL-DTL-38999*I, II, III series military electrical connectors, purchased from Huafeng Electrical Co., Ltd., Guizhou, China. The aviation wire specification is 22*AWG*, and the conductor type is 19-core stranded soft silver-plated copper wire purchased from Quanxin Cable Technology Co., Ltd., Nanjing, China. The crimping pliers (model *YJQ-W2A*) have eight crimping gears and can be used to crimp wires in the range of 12 to 26*AWG*; the electrical contact positioner (model *TH163*) can be used to assist the crimping pliers in fixing 12*#*, 16*#*, and 20*#* contacts. They were all purchased from Jingrui Instrument & Equipment Co., Ltd., Jiaxing, China.

By adjusting the crimping gear to change the feed displacement of the indenter, we can change the indentation depth of the crimping barrel. The manufacturer-provided design documents gave the indenter feed displacement corresponding to each crimping gear. Based on this, according to the outer diameter of the 20*#* electrical contact crimping barrel, the indentation depth corresponding to each crimping gear can be determined, as shown in Table 4. It can be seen that only in the 1 ~ 5*#* gear will there be an extrusion effect between the crimping barrel and the stranded conductor. Therefore, in this experiment, only crimping samples were made in the 1 ~ 5*#* gear, and five pieces were made in each gear. Figure 6b shows the crimping example.

**Table 4:** Corresponding indentation depth of 20# electrical contact crimping barrel under each crimping gear.

Gear No. (*#*)	Indentation depth (*mm*)
1	0.4709 ~ 0.5344
2	0.4201 ~ 0.4836
3	0.3693 ~ 0.4328
4	0.3312 ~ 0.3947
5	0.2550 ~ 0.3185
6	0.1661 ~ 0.2296
7	0.0772 ~ 0.1407
8	-0.0371 ~ 0.0264

### Pull-out force test of crimp terminals

The terminal pull-out force tester was purchased from Xiangbang Automation Equipment Co., Ltd., Shenzhen, China, with a maximum load of 500* N*, an accuracy of 0.1* N*, and a test range of 50* mm*.

**Figure 7:**
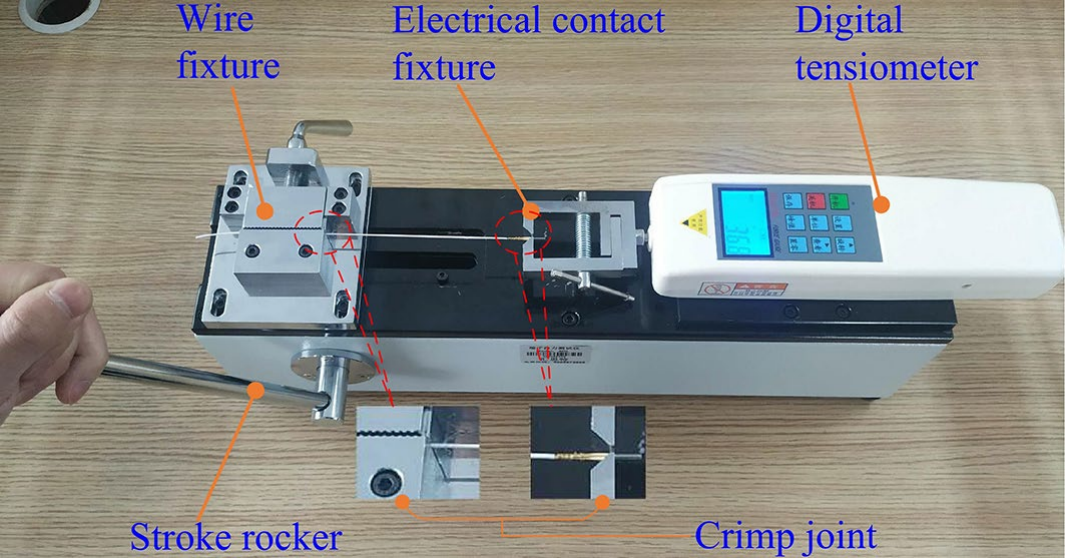
Pull-out force detection of crimp terminals.

The detection method of the pull-out force of the crimp terminal is shown in Fig. 7. After assembling the terminal pull-out force tester, fix one end of the electrical contact of the crimping sample to the right fixture and one end of the wire to the left institution, and keep the sample horizontal. Set the digital tensiometer to the "Maximum" mode, turn the stroke rocker to move the left fixture to the left at a speed of about 25* mm/min*, and record the failure type of each sample and the maximum tension value after failure.

## Results and discussion

### Mesh verification

**Figure 8:**
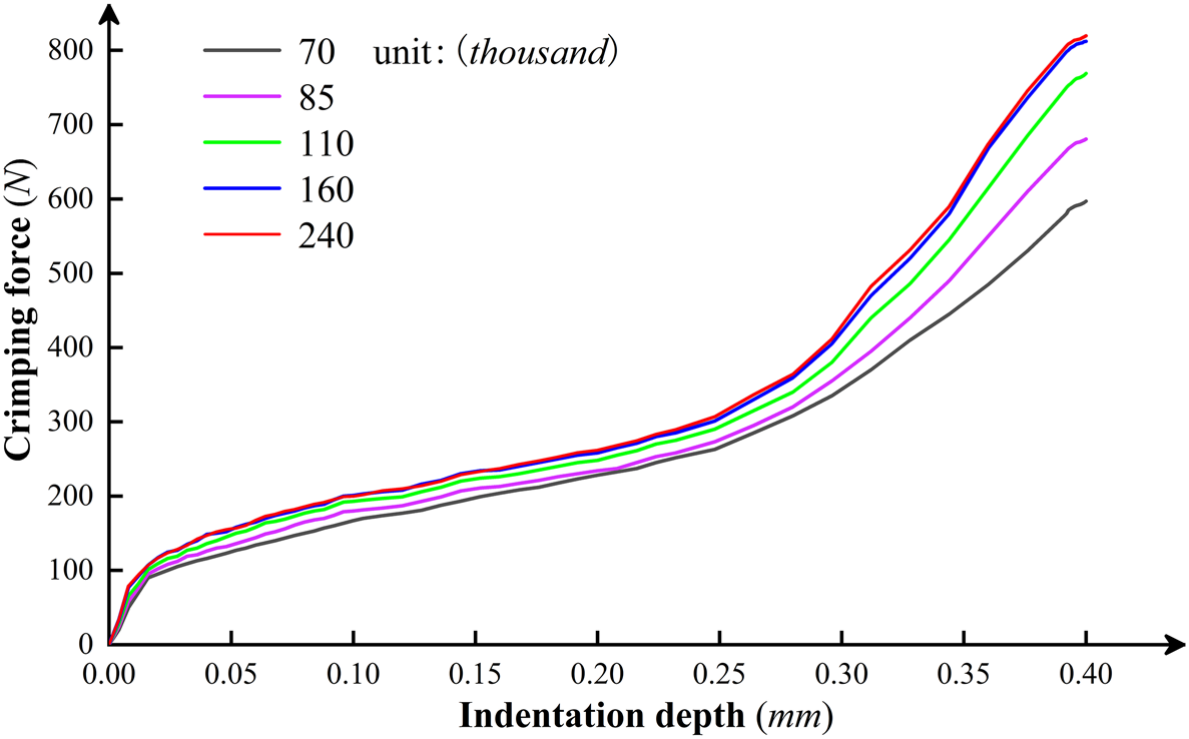
Crimping force curve as a function of the indentation depth under different numbers of mesh elements.

During the verification process, we set the indentation depths under different mesh densities to 0.4 mm, and other constraints and loading conditions were kept unchanged. In the crimping simulation, by capturing the indenter's normal contact force data acting on the crimping barrel’s surface at each time node, the crimping force curve shown in Fig. 8 as a function of the indentation depth under different numbers of mesh elements could be obtained. Generally, if the relative error between the numerical solutions at adjacent two groups of mesh element numbers is less than 5%, the effect of mesh on the results is considered acceptable. It can be seen that when the number of elements is below 160,000, the change in mesh density has a high impact on the calculation results. However, with a further increase in the number of elements, the fit of the crimping force curve is better, and the sensitivity of the calculation results to the mesh is negligible. Therefore, it is reasonable to select the mesh refinement element size corresponding to the number of mesh elements of 160,000 for the crimp simulation.

## Comparison between experiment and simulation

### Crimping effect

**Figure 9:**
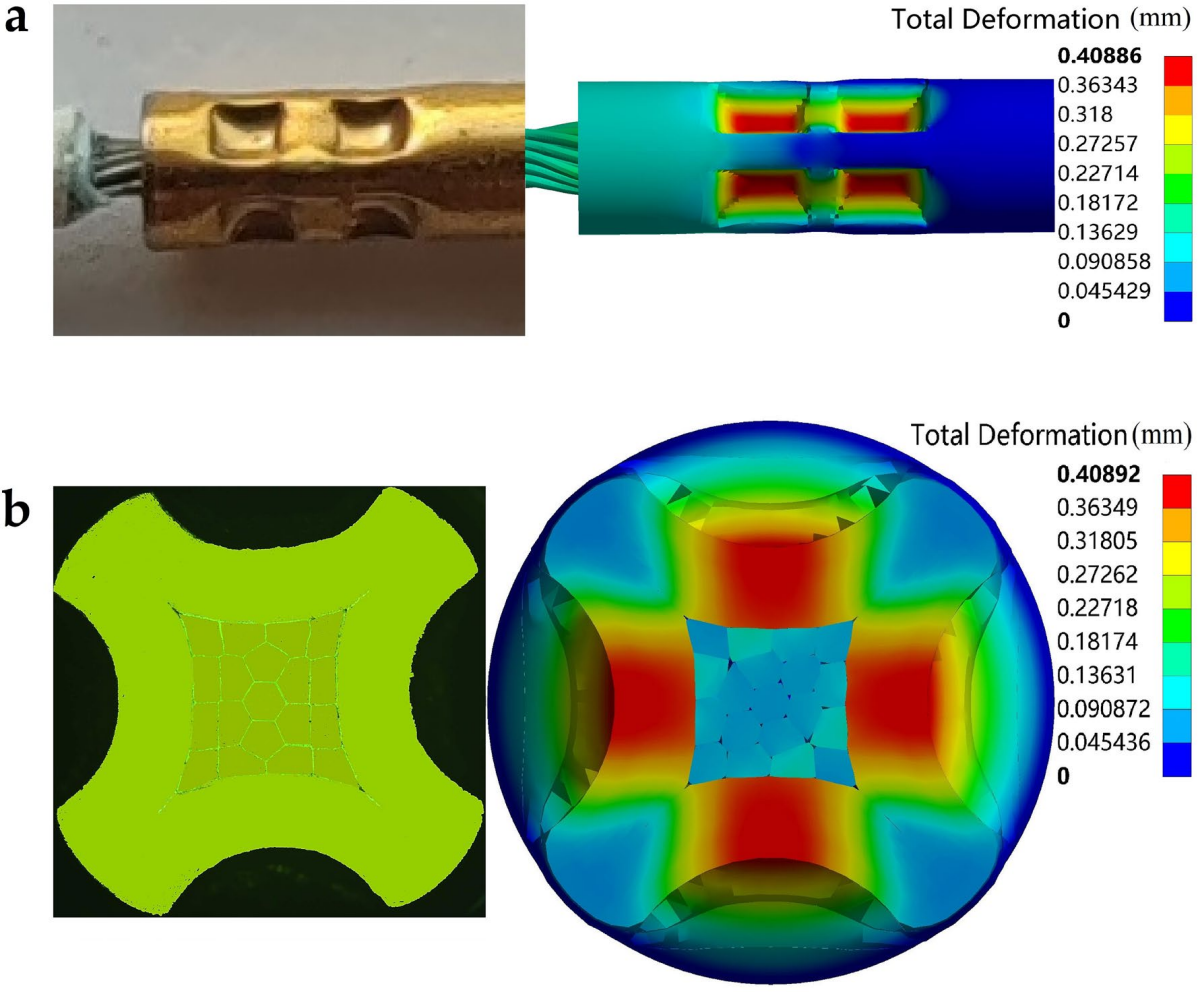
Comparison of crimping assembly geometries in experiment and simulation.

In the user manual of the crimping plier, it is recommended to select the 3*#* gear to crimp the specific combination of 20*#* electrical contacts and 22*AWG* aviation wires. Figure 9a shows the crimping simulation deformation results under the middle value (0.4* mm*) of the indentation depth range corresponding to the 3*#* gear. Compared with the real crimping assembly in the 3*#* gear, it can be seen that the geometric shapes of the two are the same. At the same time, metallographic microscopic analysis was carried out on the radial deformation of the cross-section of the crimped part of the sample, as shown in Fig. 9b. In the corresponding numerical results, although there are some slight gaps inside the compressed stranded conductor, after the load of the indenter is released, the stranded conductor and the inner wall of the crimping barrel still maintain a complete extrusion state. Therefore, these small gaps do not have a substantial impact on the subsequent simulation results of the crimp terminal pull-out force.

**Figure 10:**
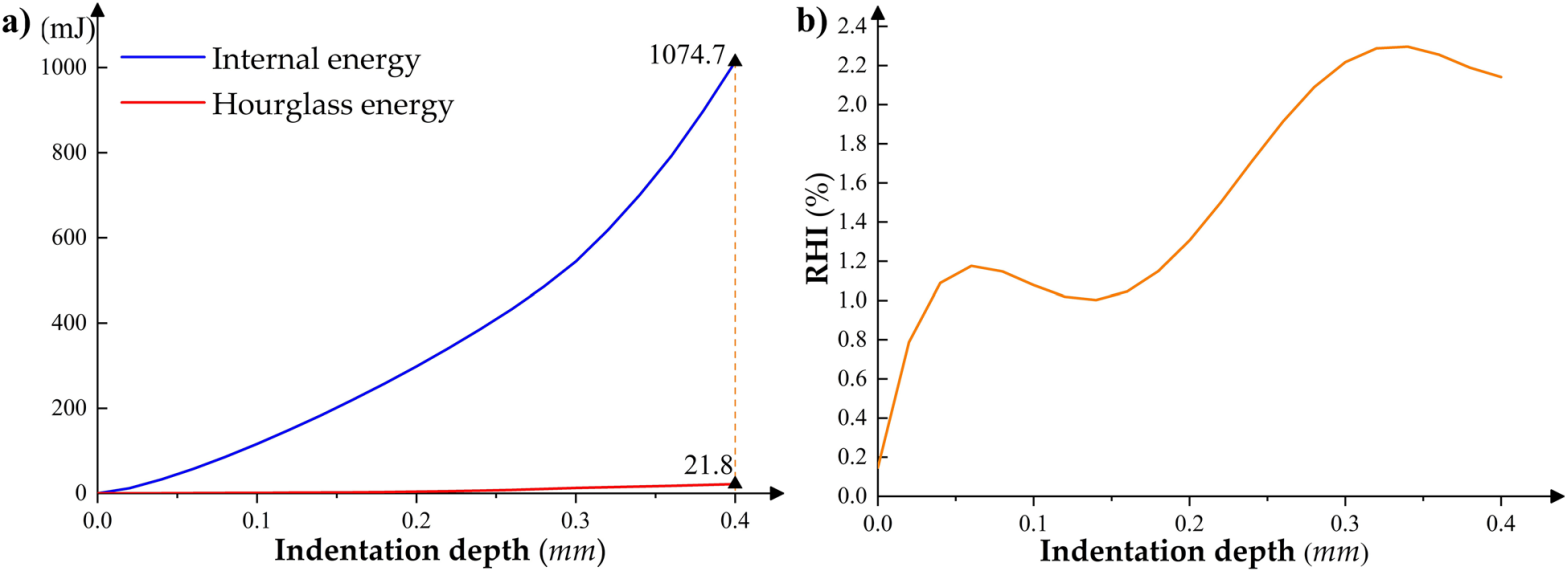
Variation of system energy with indentation depth during crimping simulation. (**a**) Internal energy and hourglass energy; (**b**) The “RHI” value as a function of indentation depth.

The ratio of hourglass energy to internal energy (RHI) can be used to examine the system's energy balance that may be disrupted by hourglass control. Several studies on elastic–plastic contact deformation simulation have given a basic standard of RHI value that can determine the computational quality^37–39^. The RHI value after the simulation should not be greater than 10%. Figure 10a shows the curves of the system’s internal and hourglass energy as a function of indentation depth during the crimping simulation. Figure 10b indicates that the RHI value of the simulation process is lower than 2.5%, and the final value is about 2%. It suggests that the system energy during the crimping simulation process is balanced, and the calculation accuracy is high. At the same time, this also verifies the rationality of the contact definition, meshing, and hourglass control type selection of the overall crimping model in Sections “Contact Definition and meshing” and “Boundary conditions and simulation parameter settings”.

#### Pull-out force and tensile failure mode of crimp terminals

According to standard *SAE-AS22520*, for the specific combination of 20*#* electrical contact and 22*AWG* aviation wire, the pull-out force of the crimp terminal shall not be less than 60* N*. Table 5 records the test values of the pull-out force and the corresponding failure mode of the crimp terminal samples under the 1 ~ 5*#* crimping gears in the experiment. When the 1*#* and 5*#* gears were selected, since the assemblies were in two states of over-crimping and under-crimping, respectively, the pull-out force of the corresponding crimping sample did not meet the standard requirements at all. For the tensile failure mode of the crimp terminal, the former showed that the stranded conductor was easily broken at the crimping part, and the latter showed that the stranded conductor slipped out of the electrical contact. In addition, when the 4# gear was selected, the failure mode was that the stranded conductor was partially broken at the crimping part, and part of the wire core slipped out of the electric contact crimping barrel. Although the pull-out force of the crimp terminal did not reach the optimal value, it still met the standard requirements.

**Table 5:** Pull-out force and failure mode of crimping samples under different crimping gears.

Gear No. (*#*)	Pull-out force (*N*)	Failure mode
1	42.7/40.5/43.6/39.7/40.1	Strands breakage
2	72.1/70.3/69.8/73.1/71.5	Strands breakage
3	83.3/82.5/81.9/81.3/82.5	Strands breakage
4	72.1/70.5/68.7/69.3/70.6	Partial breakage of strands
5	33.3/36.1/31.8/32.7/35.1	Strands slippage

**Figure 11:**
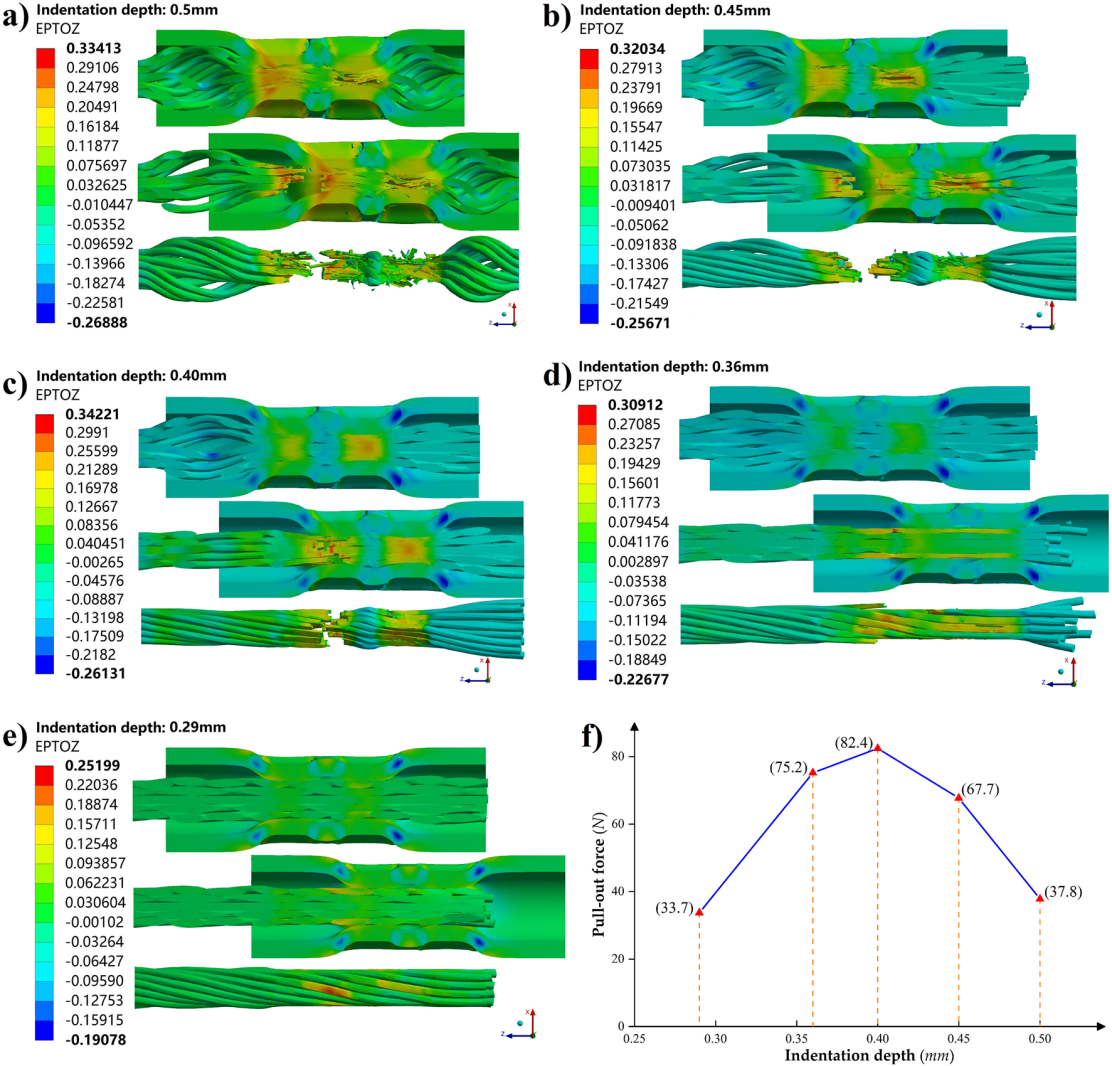
Tensile simulation results of the crimping assembly at the indentation depth corresponding to the 1 ~ 5# gear. The indentation depths are respectively taken as: (**a**) 0.5 mm; (**b**) 0.45 mm; (**c**) 0.4 mm; (**d**) 0.36 mm; (**e**) 0.29 mm. (**f**) The pull-out force of crimp terminals for each indentation depth.

Select the indentation depth range's median value (0.5* mm*, 0.45* mm*, 0.4* mm*, 0.36* mm*, and 0.29* mm*, respectively) corresponding to 1 ~ 5*#* gear for crimping simulation. Then, based on the sequential coupling method, one end of the stranded conductor of the crimping assembly model with the stress–strain results was set to a fixed state, and a certain displacement was added to one end of the electrical contact, which can realize the simulation of the pull-out force and failure mode of the crimp terminal. Here, for crimping components without apparent yield plateau, such as copper and copper alloys, the tensile failure state of the material can be approximately represented by tensile strain at break instead of elongation at break^40^. Figure 11 shows the tensile simulation results, and we can find that the numerical results at each indentation depth are in good agreement with the experimental results shown in Table 5 for the pull-out force and tensile failure mode of the crimp terminal. Among them, it is worth noting that at the indentation depth corresponding to gears 1*#* (Fig. 11a) and 2*#* (Fig. 11b), the stranded conductors are locally crushed at the crimping position, which is not advisable.

**Figure 12:**
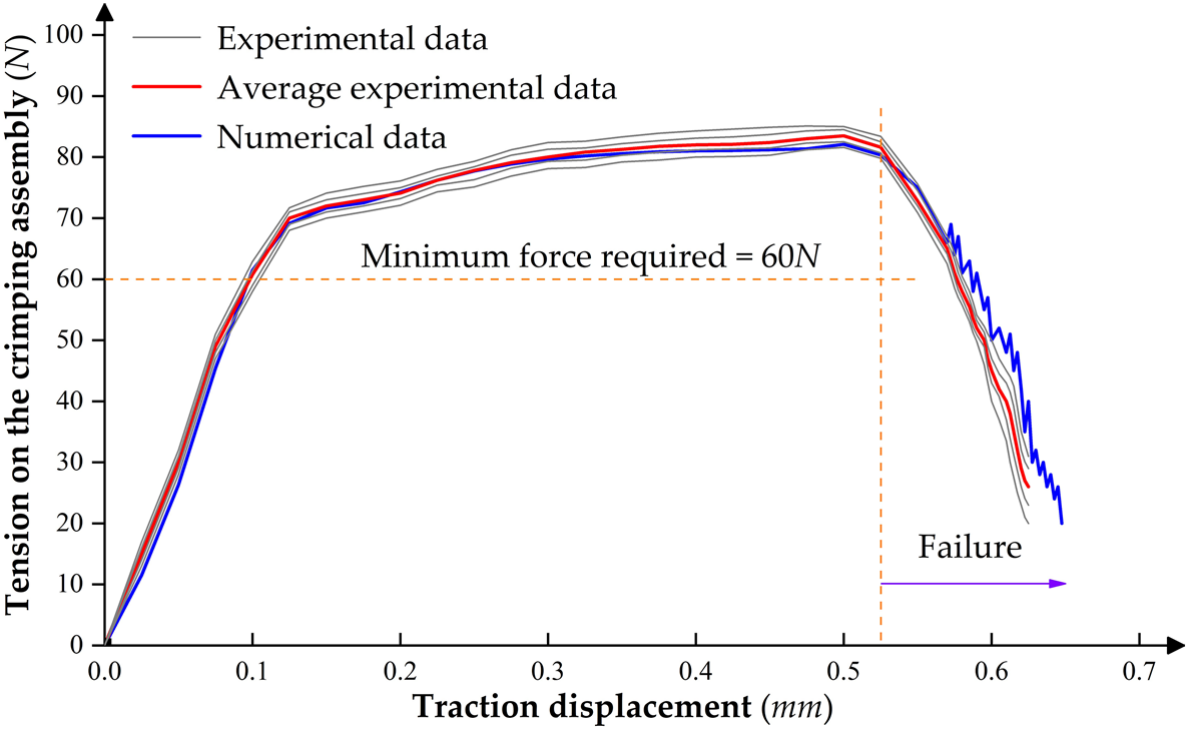
Experimental and simulated force–displacement curves for the tensile strength of the crimping assembly corresponding to the 3# crimping gear.

In the tensile simulation, we can obtain the tension of the crimping assembly by capturing the opposing force at the fixed end of the stranded conductor at each time node. Here, to further verify the reliability of the tensile simulation results of the crimping assembly, the relationship between the tension and the traction displacement in the tensile simulation results at the indentation depth corresponding to the 3*#* crimping gear was compared with the one in the experimental results. Figure 12 superimposed the force–displacement curves of the tensile simulation and multiple sets of tensile tests, and the average experimental data curve was also plotted. We can find that before the crimp terminal failed, the force–displacement trends in the practical and simulated results were the same. The overall experimental data has low dispersion, and the relative error between each group of experimental data and the average data is less than 3.4%. Moreover, the relative error between the simulation data and the experimental average data is less than 2.6%, which is enough to show that the established tensile simulation model of the crimping assembly has high computational accuracy. After failure, the stranded conductor gradually slipped out of the crimping barrel due to partial breakage, resulting in an oscillation phenomenon in the pull-out force simulation data.

### Determination of optimal indentation depth range

**Figure 13:**
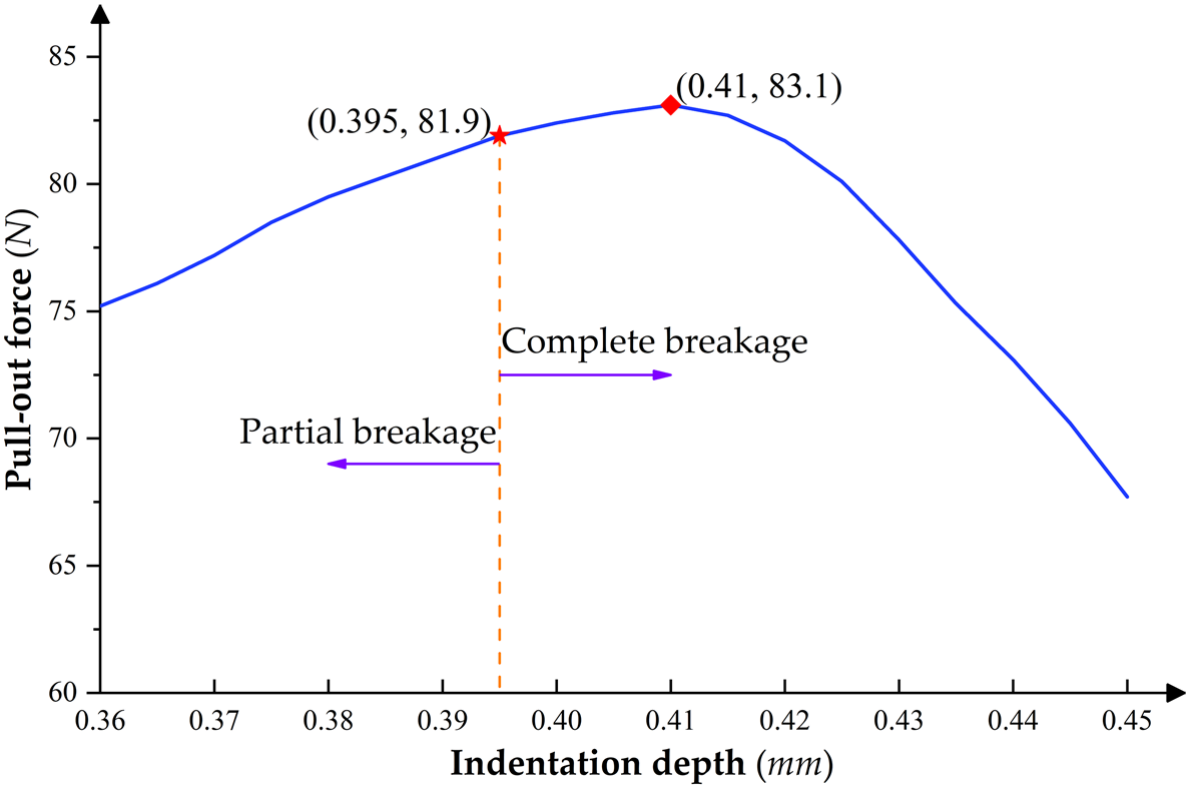
Variation of crimp terminal pull-out force and its failure mode with indentation depth.

Based on the tensile simulation results of the crimping assemblies in Section  “Pull-Out Force And Tensile Failure Mode Of Crimp Terminals”, the optimal indentation depth range for the specific combination of 20*#* electrical contacts and 22*AWG* aviation wires can be initially narrowed to within 0.36 ~ 0.45* mm*. Then, within this range, a series of crimping and assembly tensile strength simulations were carried out with 0.005 mm as the indentation depth increment, and the variation of crimp terminal pull-out force and its failure mode with indentation depth was obtained, as shown in Fig. 13. It can be seen that when the indentation depth is greater than 0.395* mm*, the stranded conductor begins to show a complete breakage phenomenon at the crimping part, and the indentation depth corresponding to the peak of the pull-out force is also in the complete breakage failure mode region. Therefore, considering the possible dimensional manufacturing errors of the crimping components and the indenter, to avoid over-crimping, the selected indentation depth should be within the range where the pull-out force is not less than 95% of the peak value, and the depth is less than the corresponding value at the peak value. The optimal indentation depth range should be 0.38 ~ 0.41* mm* for the crimping combinations considered here.

## Conclusions

This paper studies the quasi-static multi-domain contact problem of aviation electrical contact crimping by explicit dynamic finite element simulation method based on theoretical analysis. The conclusions are as follows:

1. The simulation and experimental results on the extrusion deformation of the crimping assembly showed a high degree of fit, and the RHI value of the system after the simulation was only about 2%, which verified the reliability and stability of the established crimping numerical model.
2. Based on the sequential coupling method, a tensile failure simulation was performed on a crimping assembly model with stress–strain results. The tensile failure modes in the simulation and experimental results were consistent, and the relative error of the pull-out force was only 2.6%, which showed that the established numerical model on the tensile strength of the crimping assembly has high computational accuracy.
3. For the specific combination of 20# electrical contact and 22AWG aviation wire, the pull-out force curve of the crimp terminal as a function of the indentation depth was obtained, and the optimal indentation depth range corresponding to the combination was analyzed and given.

## Data Availability

The datasets of related experiments and simulations involved in this article are available from the corresponding author upon reasonable request.
